# Acute Variceal Hemorrhage in Germany–A Nationwide Study of 65,357 Hospitalized Cases: Variceal Hemorrhage in Germany

**DOI:** 10.1155/2024/5453294

**Published:** 2024-10-24

**Authors:** Alexander Mertens, Tobias Essing, Anselm Kunstein, Christian Weigel, Johannes Bode, Christoph Roderburg, Tom Luedde, Jennis Kandler, Sven H. Loosen

**Affiliations:** ^1^Department of Gastroenterology, Hepatology and Infectious Diseases, University Hospital Düsseldorf, Medical Faculty of Heinrich Heine University Düsseldorf, Düsseldorf 40225, Germany; ^2^Department of Internal Medicine II, Marien-Hospital, Wesel 46483, Germany

**Keywords:** adverse events, AVH, EST, EVL, incidence, endoscopic therapy, in-hospital mortality, outcome, TIPS, variceal bleeding

## Abstract

**Background:** Acute variceal hemorrhage (AVH) is a frequent cause of upper gastrointestinal bleeding (UGIB) in liver cirrhosis. Most cases require urgent endoscopic intervention due to potentially life-threatening courses. Different endoscopic hemostasis techniques can be used, in particular endoscopic variceal ligation (EVL) and endoscopic sclerotherapy (EST), depending on the bleeding side (esophageal, fundal, and gastric) as well as radiological interventions (e.g., embolization and transjugular intrahepatic portosystemic shunt [TIPS]). This study aimed to investigate trends in incidence, treatment modalities, and outcome parameters, such as in-hospital mortality and adverse events in Germany.

**Methods:** We evaluated the current epidemiological trends, therapeutic strategies, and in-hospital mortality of AVH in Germany based on the standardized hospital discharge data provided by the German Federal Statistical Office from 2010 to 2019.

**Results:** A total of 65,357 AVH cases, predominately males (68.3%), were included in the analysis. The annual incidence rate (hospitalization cases per 100,000 persons) was 8.9. The in-hospital mortality was 18.6%. The most common underlying disease was alcohol-related liver cirrhosis (60.6%). The most common clinical complication was bleeding anemia (60.1%), whereas hypovolemic shock (12.8%) was the less frequent. In esophageal variceal hemorrhage (EVH), EVL was the most frequently performed endoscopic therapy, while in gastric variceal hemorrhage (GVH), EST and fibrin glue injection were the most commonly performed therapies. EVL showed the lowest in-hospital mortality (12.3%) in EVH, while EST showed favorable results (14% in-hospital mortality) in GVH. Combination therapies overall showed a higher in-hospital mortality and were more frequent in GVH. The presence of hypovolemic shock, AKI, sepsis, artificial ventilation, ARDS, bleeding anemia, hepatic encephalopathy, and male sex was associated with a significantly worse outcome.

**Conclusion:** Our study provides detailed insight into the incidence, patient-related risk factors, endoscopic treatment, and in-hospital mortality in a sizeable AVH collective in Germany. These data might help improve risk stratification and treatment strategies for AVH patients in the future.

## 1. Introduction

Liver cirrhosis is the fifth leading cause of death in adults, and one of the main reasons for cirrhosis-related morbidity and mortality is the development of acute variceal hemorrhage (AVH) [1]. Advanced cirrhosis can cause significant portal hypertension (PH), responsible for many of the complications observed in patients with cirrhosis, such as varices and ascites [2–4]. Varices can occur in different localizations through the whole gastrointestinal tract and extraintestinal sides at locations where portal veins are in contact with systemic veins [5]. Still, the most frequent and clinically relevant localizations are esophageal varices, fundal and gastric varices, or a combination [6]. In patients with liver cirrhosis, AVH is accountable for 70% of all upper gastrointestinal bleeding (UGIB) events [7], and it can be estimated that up to 30%–50% of patients with varices will experience bleeding [8]. Furthermore, AVH remains one of the most severe and potentially life-threatening complications in patients with cirrhosis and is the second most frequent decompensating event after ascites and also represents a significant economic issue [9–11]. Variceal bleeding mortality has decreased in the last 4 decades from 42% in the early 1980s [12] to the actual rates ranging between 10%–20% in most publications [13–15]. Still, there is a large spectrum with mortality rates from 6% up to > 50% depending on the patient collective, inclusion and exclusion criteria, and the type of study [16]. Only a tiny proportion of all deaths are prehospital (3%) from uncontrolled bleeding. At the same time, most of the patients die due to infection, kidney failure, hepatic encephalopathy (HE), rebleeding, or uncontrolled secondary bleeding in the first weeks after the initial episode [9, 17, 18]. AVH (and suspected AVH) usually requires urgent endoscopic examination, and an upper gastrointestinal endoscopy should be performed within 12 h according to recent guidelines to achieve hemodynamic stabilization and hemostasis and to prevent complications [7, 11, 19–21]. Endoscopic treatment of bleeding esophageal varices was initially described by Crafood and Frenckner in 1939 [22]. Different endoscopic techniques are available, such as endoscopic variceal ligation (EVL) and endoscopic sclerotherapy (EST). EVL was first described in 1988 [23]. During EVL, varices are ligated with rubber bands [20]. EST comprises injecting a sclerosing agent (sodium tetradecyl sulfate, sodium morrhuate, ethanolamine oleate, polidocanol, or absolute alcohol) via a flexible catheter with a needle tip into the variceal lumen or nearby varix. EST in AVH can also be done with tissue adhesives such as n-butyl-2-cyanoacrylate (Histoacryl) or isobutyl-2-cyanoacrylate (bucrylate). Tissue adhesives and fibrin glue (a combination of thrombin and fibrinogen), which are both injected (strictly intravascular) into esophageal or gastric varices, lead to immediate vessel obliteration by polymerization and hardening [24–27]. EST can be used in AVH and primary or secondary bleeding prevention [7, 20, 28]. EVL is currently considered the first-line endoscopic therapy to prevent and treat esophageal variceal hemorrhage (EVH). EST seems to have a slightly lower rate of primary hemostasis and higher rates of complications in EVH [7, 21, 29, 30]. Nevertheless, there is no clear advantage regarding overall mortality comparing EVL and EST. Still, nowadays, EST is mainly limited to actively bleeding cases in which banding is not feasible or has failed and in gastric variceal hemorrhage (GVH) [29, 30]. In GVH, the current guidelines recommend cyanoacrylate injection (CI) as the most common tissue adhesive for acute gastric (cardiofundal) variceal (gastroesophageal varices type-2 [GOV-2] and isolated gastric varices type-1 [IGV-1]) hemorrhage and as an alternative to EVL in patients with gastroesophageal varices type-1 (GOV-1)-specific bleeding [21]. Transjugular intrahepatic portosystemic stent shunt (TIPS) is not recommended as primary prophylaxis of variceal bleeding but can be used as a rescue therapy option in AVH if bleeding is not controllable via endoscopic therapy and preemptive in secondary prevention after bleeding within 24–72 h, especially in high-risk situations for rebleeding [7, 11, 31]. Other endoscopic procedures such as balloon tamponade, esophageal stent implantation, endoscopic clipping, or argon plasma coagulation (APC) are not the standard of care (SOC). They are reserved for uncontrolled bleeding or as a bridge to TIPS. Detailed population-based data about the incidence, risk factors, SOC treatment, and in-hospital mortality of AVH are missing. Therefore, this study aimed to give a detailed insight into these aspects by including all common endoscopic and nonendoscopic therapeutic strategies over a long observational period in Germany, the most populous country in the European Union.

## 2. Materials and Methods

### 2.1. Study Design

The present study represents a retrospective analysis of epidemiological trends, in-hospital mortality, comorbidities, endoscopic therapies, and risk factors for poor AVH survival in Germany. The Federal Statistical Office of Germany (Wiesbaden, Germany) provided the standardized hospital discharge data used for the analyses from 2010 to 2019. In 2020, the Federal Statistical Office and the University Hospital Duesseldorf signed a contract for remote data analysis. Due to the complete anonymization of patient information, no additional ethics approval was necessary.

### 2.2. Patient Eligibility Criteria and Variables

The study population was identified via the specific primary diagnosis of the respective hospital stay using the ICD-10 code for esophageal and gastric variceal bleeding due to liver disease (I85.0 and I98.3). Comorbidities were identified by using the following ICD codes: alcoholic fatty liver disease (K70.9), alcoholic hepatitis (K70.1), alcoholic fibrosis and sclerosis (K70.2), alcoholic liver cirrhosis (K70.3), alcoholic liver failure (K70.40, K70.41, K70.42, and K70.48), toxic liver disease without cholestasis (K71.0), toxic liver disease with necrosis (K71.1), toxic liver disease with other affection (K71.8 and K71.88), toxic liver disease not specified (K71.9), toxic liver disease with acute or acute on chronic or chronic hepatitis or not specified hepatitis (K71.2, K71.3, K71.4, K71.5, and K71.6), toxic liver disease with cirrhosis (K71.7), liver fibrosis and sclerosis (K74.0, K74.1, and K74.2), liver cirrhosis (K74.4, K74.5, and K74.6), primary biliary cirrhosis and biliary cirrhosis not specified (K74.3), hemochromatosis (E83.1), chronic inflammatory hepatitis (K75.9), other liver diseases not specified (K76), acute hepatitis A (B15), acute hepatitis B (B16), acute viral hepatitis not specified (B17), chronic hepatitis B (B18), viral hepatitis not specified (B19), and hepatocellular carcinoma (C22.0). The Child–Pugh stadium (A–C) was identified via the ICD codes (K74.70, K74.71, and K74.72). Specific endoscopic treatment approaches were identified using the following OPS codes: sclerotherapy of fundal varices (5-449.03), band ligation of fundal varices (5-449.83), gastric application of absorbent substances (5-449.t3), gastric fibrin glue injection (5-449.e3), sclerotherapy of esophageal varices (5-429.1), band ligation of esophageal varices (5-429.a), esophageal endoloop application (5-429.c), esophageal clipping (5-429.d), and esophageal fibrin glue injection (5-429.e). Other treatment approaches were selective embolization with embolizing fluids, visceral vessels (8-836.9a), selective embolization with particles, visceral vessels (8-836.ka), and TIPS (8-839.87, 8-839.88, 8-839.89, and 8-8398.a).

Patients with organ complications were identified by the following secondary diagnosis: ascites (R18), acute renal failure (N17), hepatorenal syndrome (K76.7), hypovolemic shock (R57.1), bleeding anemia (D62), HE (K72.71!, K72.72!, K72.73!, K72.74!, and K72.79!), sepsis (A32.7, A39.2, A39.3, A.40.0, A40.1, A40.2, A40.3, A40.8, A40.9, A40.1, A41.1, A41.2, A41.3, A41.4, A41.51, A41.52, A41.58, A41.8, A41.9, A42.7, A26.7, A0.21, A20.7, and B37.7), and adult respiratory distress syndrome (ARDS) (J80). Furthermore, subsequent clinical and demographical variables were assessed, namely, gender, age, duration, and necessity of mechanical ventilation, in-patient department, number of cases per hospital, and federal state of treatment. In-hospital mortality was defined as the proportion of patients whose discharge status was designated as “death.”

### 2.3. Statistical Analysis

We performed all statistical analyses via remote data access at the Federal Statistical Office (Destatis; Wiesbaden, Germany) using the statistical program SPSS (IBM SPSS Statistics, Version 28.0 Armonk, NY: IBM Corp., USA) and the spreadsheet program Excel (Microsoft Corporation, Redmond, WA, USA). Cross-tabulations were generated for the analysis of descriptive data. Welch's *t*-test, the chi-squared test, and Fisher's exact test were applied to assess differences in the frequency and type of treatment approaches and in-hospital mortality. Univariate and multivariate regression and Pearson's correlation coefficient were calculated to analyze correlations. All statistical tests were two-sided, and a *p* value < 0.05 was considered significant.

## 3. Results

### 3.1. Study Population

**Figure 1:**
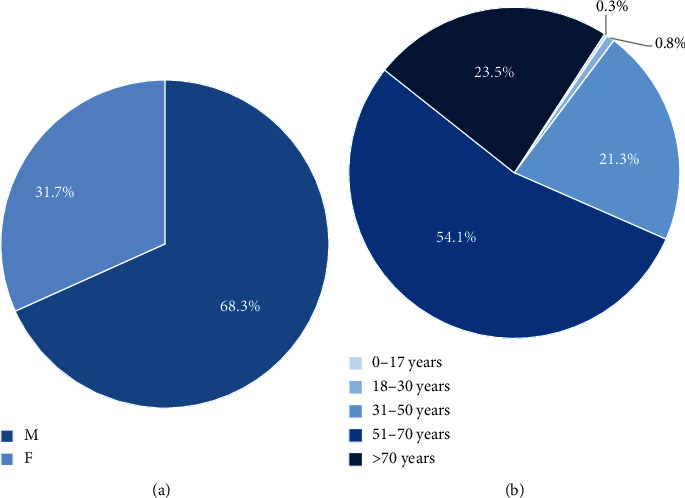
Study population: (a) sex distribution and (b) age distribution.

A total of 65,357 AVH cases were included in the analysis (Table 1). Most cases were male patients (68.3%; Table 1 and Figure 1(a)) and those over the age of 50 (77.6%, Table 1 and Figure 1(b)). The mean age was 60.3 (SD ± 12.9). 0.3% (*n* = 172) of the cases were minors (below 18 years of age) (Table 1 and Figure 1(b)).

**Figure 2:**
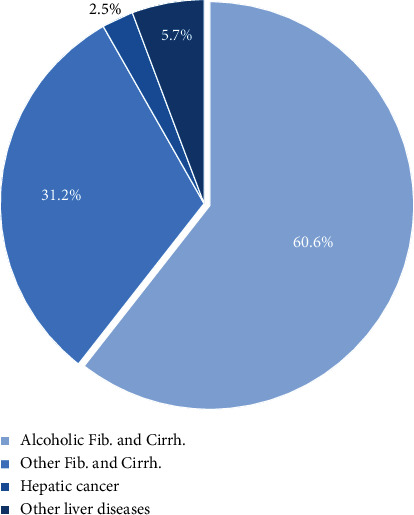
Study population, underlying etiology for liver disease.

**Figure 3:**
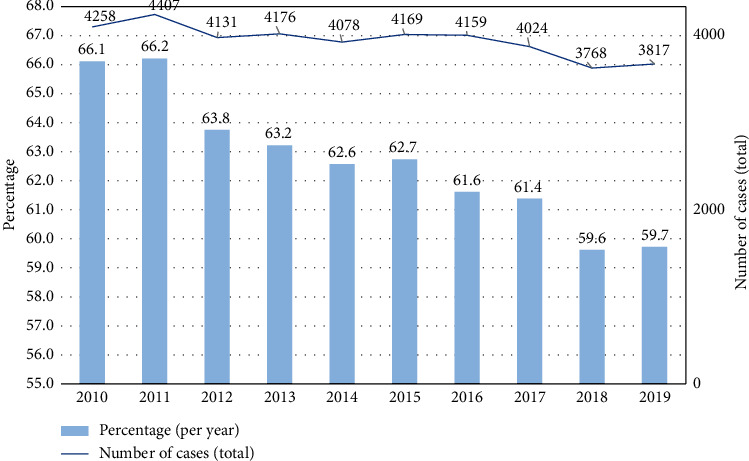
Study population, alcohol-related acute variceal hemorrhage; total number of cases per year between 2010 and 2019 (solid line), right *y*-axis. Cases are as percentages per year between 2010 and 2019 (bars), on the left *y*-axis and the *x*-axis: year.

The most common underlying etiology of fibrosis and cirrhosis was alcoholic liver disease (*n* = 39,589; 60.6%), while other etiologies (e.g., viral and toxic cirrhosis) combined contributed to 31.2% of the cases (*n* = 20,385) (Table 1 and Figure 2). Other liver diseases without fibrosis or cirrhosis (*n* = 3740; 6.6%) and HCC (*n* = 1643; 2.5%) played a minor role (Table 1 and Figure 2). The total number of alcohol-related liver damage decreased over time as well as the proportion (Figure 3).

**Figure 4:**
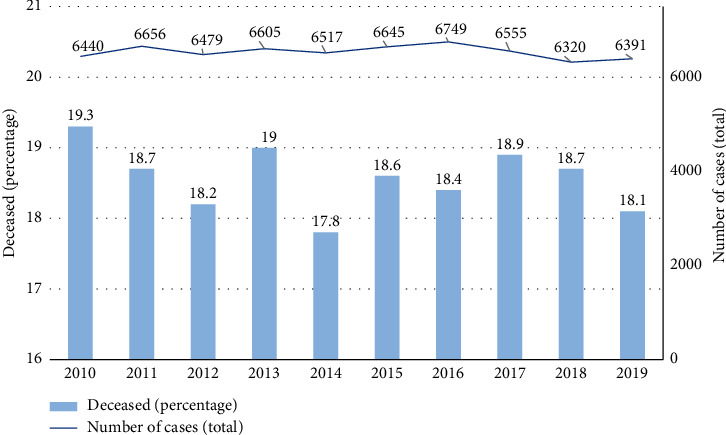
Total number of acute variceal hemorrhage cases per year between 2010 and 2019 (solid line), right *y*-axis. Mortality per year between 2010 and 2019 (bars), left *y*-axis; *x*-axis: year.

The average annual number of AVH cases in Germany was 6536 and did not differ significantly during the observational period (SD ± 125.4; lowest count: 6320 in 2018; highest count: 6749 in 2016) (Table 2 and Figure 4).

### 3.2. In-Hospital Mortality

**Figure 5:**
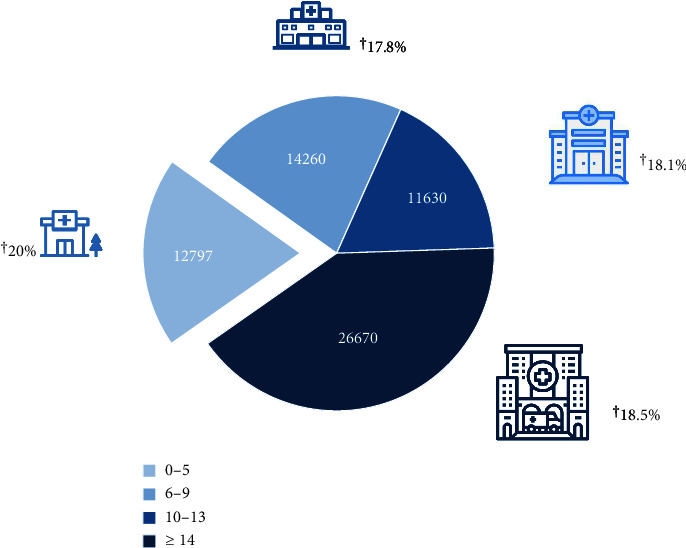
Distribution of acute variceal hemorrhage cases (total number, inside pie chart) concerning the volume of the centers (cases per hospital/year, label below the graph) and mortality in each group (as a percentage, next to the hospital icons).

The overall in-hospital mortality was 18.6% (Table 2). The in-hospital mortality did not significantly differ during the observation period (Table 2). We next subdivided the hospitals into four groups depending on the quartiles of the annual number of AVH cases. Age was associated with higher in-hospital mortality (odds ratio [OR] per year: 1.02 [95% CI: 1.02–1.03]). Female sex was associated with lower in-hospital mortality (OR: 0.91 [95% CI: 0.86–0.96]). There were significant differences in in-hospital mortality concerning the annual case volume of variceal hemorrhage per hospital (Table 2 and Figure 5). Hospitals with the lowest case number (1–5 cases/year) reported had in-hospital mortality of 20% vs. combined 18.2% in the other three groups (6–9 cases per year, OR: 0.78 [95% CI: 0.72–0.85]; 10–13 cases per year, OR: 0.77 [95% CI: 0.71–0.84]; ≥ 14 cases/year, OR: 0.58 [95% CI: 0.54–0.62], *p* < 0.001) (Table 2 and Figure 5).

### 3.3. Regional Distribution

**Figure 6:**
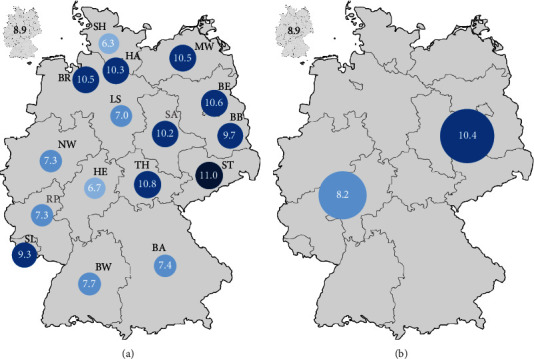
Incidence of AVH in Germany. (a) Incidence of AVH per 100,000 residents per year and federal state; left top corner: nationwide incidence between 2010 and 2019 (8.9). (b) Incidence of AVH in the New Federal States without Berlin (right, deep blue) and in the Old Federal States including Berlin (left, pale blue); left top corner: nationwide incidence between 2010 and 2019. BB: Brandenburg, BE: Berlin, BW: Baden-Württemberg, BA: Bavaria, HE: Hesse, BR: Bremen, HA: Hamburg, MW: Mecklenburg–Western Pomerania, LS: Lower Saxony, NW: North Rhine–Westphalia, RP: Rhineland-Palatinate, SH: Schleswig-Holstein, SL: Saarland, SA: Saxony, ST: Saxony-Anhalt, and TH: Thuringia.

Considering the incidence rate of AVH in Germany, most cases were registered in North Rhine–Westphalia (*n* = 12,897; 19.7%) (Table 2). Bavaria observed 9411 (14.4%) cases during the observation period. The lowest number was recorded in Bremen (*n* = 702; 1.1%) (Table 2). Based on the population numbers, Saxony had the highest incidence rate (11/year/100,000 residents) followed by Thuringia (10.8/year/100,000 residents) and Berlin (10.6/year/100,000 residents) (Figure 6). Schleswig-Holstein (6.3/year/100,000 residents) recorded the lowest incidence rate (Figure 6). This results in a 1.7-fold higher incidence of AVH between the state with the highest incidence (Saxony) and the state with the lowest incidence (Schleswig-Holstein). The national average was 8.9 cases/year/100,000 residents (Tables 2 and 3). Significant differences existed between the New Federal States (incidence rate 10.5/year/100,000 residents; without Berlin 10.4/year/100.000 residents) and the Old Federal States (8.2/year/100,000 residents) (Table 2 and Figure 6).

### 3.4. Adverse Events and Their Impact on In-Hospital Mortality

The most frequent complication was bleeding anemia (*n* = 39,295; 60.1%) followed by HE (*n* = 11,956; 18.3%) (Table 1). Acute deterioration of kidney function was common with 17.4% (*n* = 11,352), of which hepatorenal syndrome accounts for 51.7% (*n* = 5866). Hypovolemic shock (*n* = 8373; 12.8%) and artificial ventilation (*n* = 10,636; 16.3%) were frequent, while sepsis (*n* = 2466; 3.8%) was rare (Table 1). 16.3% of all AVH cases were artificially ventilated. Since 50.3% of the ventilation could be terminated within the first 48 h, artificial ventilation was mostly short (48–168 h: 26% (*n* = 2766); > 168 h: 23.7% (*n* = 2524)) (Table 1). ARDS was rare, with 444 cases (0.7%). 41.4% of the cases (*n* = 27,066) had ascites. The presence of hypovolemic shock (mortality of 48.9% vs. 12.3%, OR 2.76 [95% CI: 2.57–2.96], *p* ≤ 0.001), hepatorenal syndrome (HRS) (56% vs. 13%, OR 4.15 [95% CI: 3.83–4.49], *p* ≤ 0.001), acute kidney injury (AKI) (55.2% vs. 13.2%, OR 2.59 [95% CI: 2.4–2.79], *p* ≤ 0.001), HE (36.2% vs. 12.8%, OR 1.74 [95% CI: 1.64–1.86], *p* ≤ 0.001), ascites (25.4% vs. 12.1%, OR 1.16 [95% CI: 1.09–1.22], *p* ≤ 0.001), sepsis (64.7% vs. 14.4%, OR 2.19 [95% CI: 1.94–2.46], *p* ≤ 0.001), artificial ventilation (57.4% vs. 9.9%, OR 6.15 [95% CI: 5.76–6.57], *p* ≤ 0.001), ARDS (79.7% vs. 15.4%, OR 2.58 [95% CI: 1.94–3.44], *p* ≤ 0.001), and male sex (OR 1.1 [95% CI: 1.04–1.16], *p*=0.001) significantly increased the in-hospital mortality in multivariate analysis (Table 4).

### 3.5. Endoscopic and Nonendoscopic Therapies

Endoscopic and nonendoscopic therapies were used in 56,807 (87.1%) cases. In 8442 cases, no specific treatment (12.9%) was performed or documented. Regarding EVH, EVL was the most commonly performed endoscopic therapy (*n* = 39,499; 84.3% of all endoscopic EVH therapies) followed by injection (*n* = 3790; 8.1%). EST (*n* = 1276; 2.7%), tamponade (*n* = 1283; 2.7%), clipping (*n* = 614; 1.3%), and stenting (*n* = 373; 0.8%) were the less commonly applied techniques (Table 3).

In GVH, injection (*n* = 3199; 39.7% of all therapies) and EST (*n* = 2564; 31.8%) accounted together for 71.5% of all therapies, while clipping (*n* = 1571; 19.5%), EVL (*n* = 583; 7.2%), and the use of absorbent substances (*n* = 132; 1.6%) as well as TIPS (*n* = 18; 0.2%) played a minor role (Table 3). TIPS was performed in 1502 cases, and radiologic intervention (embolization) was performed in 511 cases (Table 3).

Combined endoscopic therapies were applied in 7823 cases (EVH: *n* = 6481; GVH: *n* = 1342) of all AVH cases (Table 3). In principle, combination therapies were used more often in GVH than in EVH (16.6% vs. 13.8%, *p*  <  0.001). Regarding EVH, the predominantly used combined approach was a combination of injection and EVL in 2126 cases, followed by EVL + EST in 1868 cases (Table 4). EVL was the most common combination therapy used in 5465 (84.3%) cases. TIPS was used in 722 cases as secondary therapy to EVL (Table 3). Concerning GVH, injection + clipping (*n* = 524) and injection + EST (*n* = 468) were the most frequent combination therapies (Table 3).

Regarding endoscopic therapeutic procedures and in-hospital mortality, EVH was basically associated with lower mortality than GVH (16.1% vs. 19.1%, *p* ≤ 0.001) (Table 3). In EVH, EVL had the lowest mortality (12.3%) followed by EST (20.5%), while clipping (27.9%) and injection (30.8%) went along with increased in-hospital mortality. The highest in-hospital mortality in EVH had stenting (63.5%) and tamponade (65.9%) (Table 3).

Regarding the mortality of combination therapies in EVH, there was significantly higher mortality (30.5%) compared to single-modality therapies (16.1%), *p* ≤ 0.001. EVL-based combination therapies had an in-hospital mortality of 29.5%. There were no significant differences in the in-hospital mortality between EVL + injection (32.5%) vs. EVL + clipping (31.9%), *p*=0.59, vs. EVL + EST (29.9%), *p*=0.27 (Table 3). EVL + TIPS (21.2%), *p* ≤ 0.001, and EVL + embolization (15.3%), *p* ≤ 0.001, had a lower in-hospital mortality compared to the every other (endoscopic) therapy in combination with EVL (Table 3).

Injection + clipping (39.7%) was associated with increased mortality, *p*=0.001, compared to EVL-based combination therapies. Clipping + EST (37.9%) and EST + embolization (35.7%) tended to higher in-hospital mortality than EVL combination therapies. Still, the results were not significant due to the comparatively low number of cases in both groups (*p*=0.13 resp. *p*=0.71) (Table 3). Regarding EST, the combination therapy with TIPS (26.3%) showed the lowest in-hospital mortality. EST + clipping (37.9%), EST + embolization (35.7%), and EST + injection (32.3%) were associated with higher mortality, but the results were not statistically significant (*p*=0.18, *p*=0.59, and *p*=0.4, respectively) (Table 3).

Regarding GVH and endoscopic therapies, the use of absorbent substances went along with the highest in-hospital mortality (37.9%). EVL (16.3%) and EST (14%) had comparable in-hospital mortality (*p*=0.14) and each significantly lower in-hospital mortality compared to clipping (23.4%) and injection (22.1%), *p* < 0.001 (Table 3). There was no difference between injection (22.1%) and clipping (23.4%), *p*=0.32) (Table 3).

Combination therapies in GVH were, in general, associated with higher in-hospital mortality compared to single-modality treatments (21.8% vs. 19.1%, *p* < 0.001), with the highest rates in injection + absorbent substances (37.5%) and injection + clipping (24.6%) (Table 3). Injection + EST (16.9%) showed comparatively low in-hospital mortality compared to EST alone (14%), *p*=0.78 (Table 3).

## 4. Discussion

The present nationwide study analyzed more than 60,000 cases of EVH and GVH over a period of 10 years in Germany. A recent population-based study from the United States reported 191 hospitalizations due to AVH (only EVH)/per one million residents/per year in 2011, which is more than double the average incidence we reported between 2010 and 2019, with 8.9 AVH (esophageal + stomach bleeding site)/per 100,000 residents/per year and a total of 65,357 cases between 2010 and 2019 [32]. Our data are in line with another more recent population-based study, reporting an average number of AVH (only EVH) hospitalizations per year in the United States of 29,600 between 2011 and 2018, leading to an incidence of around 9.4/100.000 residents/year [33]. Neither mentioned study reported other variceal hemorrhage localizations (e.g., stomach). Population-based data about AVH in stomach/fundal varices are generally largely lacking. Our data are the first to describe esophageal AVH in the general population in a Western European state. The mean number of AVH cases in Germany in our study was 6536/year and did not differ substantially over the years. Comprehensive recent data regarding AVH incidence are missing, possibly due to the usually short observational time of the studies and mostly single-center experiences. A former US study reported hospitalization rates of bleeding varices between 1988 and 2002 [34]. A more recent study from 2019 found an increased rate of esophageal varices in hospitalization discharges between 2001 and 2011 (+138%), while on the other hand, the number of esophageal variceal bleeding cases remained nearly stable (+7%) [32]. Another US study found a decreased incidence of EVH in hospitalized cirrhotic patients between 2002 and 2012. The authors attributed the decline to the effectiveness of primary and secondary prevention [35]. Our more recent data with a stable number of cases may reflect that during our observational period (2010–2019), no fundamental changes in primary or secondary prevention were made (e.g., EVL; nonselective beta-blocker therapy). Furthermore, it can be stated that there was no relevant change in the prevalence of liver cirrhosis in Germany (and Central Europe as well) as the main factor for variceal bleeding: Despite the increasing rates of nonalcoholic fatty liver disease (NAFLD), alcohol consumption remained by far the dominant cause of cirrhosis in Germany in the 2010s and the proportions of people with cirrhosis due to alcohol consumption decreased so that overall there is no significant change in cirrhosis prevalence between 2010 and 2019 to assume [36–38]. Indeed, we could show a decrease in AVH due to alcohol-related liver damage of 10.4% during the observational period. However, alcohol consumption and its disease sequel (alcoholic fibrosis and cirrhosis) were still the most common reasons for AVH. Other etiologies of fibrosis and cirrhosis (NAFLD, chronic viral and autoimmune hepatitis, acute nonalcoholic liver failure, and not specified liver failure) combined for 31.2% of the cases, while hepatic cancer (2.5% of the cases) was a rare comorbidity in AVH in our study. We cannot rule out miscoding because of the German coding system's imprecise definition of harmful or chronic alcohol abuse, which could cause harmful or chronic alcohol abuse to be underestimated as the primary cause of advanced liver damage. Further analyses should examine the incidence over time to confirm or rule out our findings. Nevertheless, we anticipate a legitimate decline in the incidence in the recent past because of our study's lengthy observation period of 10 years and the consistent and steady decline over the years. We assume a transferability for different populations, at least in Western Europe, because the German population is relatively representative for other Western European countries in terms of potential risk factors.

There were comparatively large regional differences between the Federal States in Germany, with the highest incidence rates in the New Federal States (the New Federal States without Berlin: 10.4; the Old Federal States with Berlin 8.2). Although our data do not allow us to identify the underlying cause of a higher incidence of AVH in the New Federal States, it is worth noting that the daily intake of alcohol (median 12.6 g vs. 10.6 g/day) and the proportion of people with alcohol consumption above 20 g/day, as well as the number of direct alcohol-related deaths, are notably higher in the New Federal States compared to the Old Federal States [39]. Furthermore, regional differences in the development and mortality rate of liver cirrhosis across the different federal states of Germany, with higher hospitalization rates and higher in-hospital mortality rates, were reported [36]. These differences may be further explained by social and economic reasons in the New Federal States [40, 41]. At the same time, nationwide data regarding the geographical distribution of other relevant factors (especially the stage of liver disease, according to the Child–Pugh score) are missing. We have to state that our data represent hospital discharge data. Thus, we cannot rule out underestimating the incidence of AVH in Germany (for example, due to outpatient cases and preclinical and subclinical/self-limiting cases without endoscopic investigation). Nevertheless, due to the usually severe and clinical overt course of AVH, it is reasonable to expect a low proportion of missed cases.

Most of the cases in our study were male (68.3%), and the average age was 60.3 years, which is both in line with other data [6, 23, 42]. The most frequent complication in our study was bleeding anemia in more than half of all cases. Systematic analysis of the prevalence of anemia in AVH is largely lacking. It is essential to state that anemia in cirrhosis is common–despite the absence of AVH: Portal hypertensive gastropathy, higher incidence of duodenal ulcer and ulcer-related bleeding in cirrhosis, nutritional deficiencies, hypersplenism secondary to PH, and direct alcohol-related bone marrow effects contribute to this [43, 44]. While bleeding anemia is frequent, hypovolemic shock was less common (12.8%) in our study but slightly higher than that other authors stated in smaller series: Ratio et al. described hypovolemic shock in 6.7% of all patients, while Bilal et al. reported a proportion of 9.9% [45, 46], whereas a single-center study from Saudi Arabia reported hypovolemic shock in 17.6% of the cases [47]. A recent meta-analysis showed a prevalence of 25%, but it was not discriminated between hemodynamic instability and hypovolemic shock by definition.

Sepsis was rare in our study data (3.8%). According to the current guidelines, there is a strong recommendation for antibiotic prophylaxis for all patients with advanced chronic liver disease (ACLD) and AVH [21]. We must state that we did not record the proportion of antibiotic therapies. Still, our data represent the clinical practice in Germany. It should be assumed that the vast majority of patients received antibiotic therapy: A recent multicenter study by Martinez et al. reported a proportion of 93.6% receiving antibiotic prophylaxis in AVH, of which 19.3% of the patients developed bacterial infection, primarily respiratory tract infections [48]. The authors did not discriminate between bacterial infection and sepsis. Data about sepsis in AVH are generally missing, possibly due to different clinical definitions over time. Most of the published data focus on bacterial infections.

Another known complication of AVH is HE. We report a total of 11,956 (18.3%) cases of HE in AVH, which is lower than the previously published data: Rudler et al. reported an incidence of 38% in a small AVH cohort (2138 patients) [49]. It is essential to mention that subclinical HE cases may be present and may be overlooked (covert HE), especially in the intensive care setting. Furthermore, the rate of correctly diagnosed hepatic encephalopathies depends on the diagnostic tool's sensitivity (e.g., psychometric examination, laboratory findings, and critical flicker frequency) [50, 51].

16.3% of the patients in our study underwent artificial ventilation in more than half of the cases short (< 48 h duration). This may represent the clinical practice in patients with massive bleeding, hematemesis, and the presence of overt HE in AVH, as airway protection with endotracheal intubation and mechanical ventilation is essential, at least peri-interventional for endoscopic intervention [52]. On the other hand, more urgent and severe clinical courses in AVH are more common than in other causes of UGIB [53, 54]. Koch et al. and Tang et al. reported a rate of artificial ventilation in AVH in a single-center hospital of 67.7%, respectively 60% in both small single-center studies, and these studies were included in meta-analyses about artificial ventilation in AVH [55, 56]. None of the other studies in this meta-analysis discriminated between different causes of UGIB. ARDS was very rare in our study, with a total of only 444 cases (0.7%), and may reflect prolonged intensive care stay due to secondary complications after AVH. To our knowledge, no published data about ARDS secondary to AVH exist.

Ascites was frequent in nearly half of the cases in our study (41.4%). Ascites is the most common decompensation event followed by AVH [57]. A single-center study revealed ascites in 74.4% of the AVH cases [47]. The high proportion of ascites in our study may reflect the high proportion of advanced liver cirrhosis as a crucial factor for AVH. Still, due to the retrospective data, this remains speculative since we could not discriminate the proportion of pre-existing ascites vs. secondary ascites after AVH and the stadium of liver cirrhosis via the Child–Pugh classification or MELD score.

Regarding the in-hospital mortality, AVH remains one of the most severe and potentially life-threatening complications in patients with cirrhosis [9–11]. Overall, the in-hospital mortality rate in our study was 18.6%, with more than 12,000 AVH-related deaths. AVH mortality has decreased in the last 4 decades from 42% in the early 1980s [12] to the actual rates ranging between 10%–20% in most publications [13–15]. Still, there is a large spectrum with mortality rates from 6% up to > 50% depending on the patient collective, inclusion and exclusion criteria, and the type of study [16]. It is important to mention that only a minority die from uncontrolled bleeding, while most of the patients die due to adverse events. Regarding adverse events of AVH, the presence of hypovolemic shock, HRS, AKI, HE, ascites, sepsis, artificial ventilation, ARDS, and male sex were independently associated with higher in-hospital mortality in multivariate analysis. It is essential to mention that the published data regarding complications of AVH is usually heterogeneous with broad distribution, depending on the respective collective. Nevertheless, hypovolemic shock, kidney failure (HRS and AKI), sepsis, and artificial ventilation are the generally accepted independent risk factors of mortality in AVH, and our data are basically in line with the previously published data [47, 58, 59]. HE is an accepted independent mortality-associated factor in liver cirrhosis [60]. On the other hand, the role of HE in AVH remains largely unclear: Mandal et al. showed higher mortality in AVH and HE, but the results failed to show significance in multivariate analysis [61]. Our data underline the role of HE in AVH and may help to investigate further potential benefits in early screening and treatment of HE. We have to state that we were only able to capture the in-hospital course, and the mid-to-long-term consequences of the reported adverse events after AVH in our collective remain unclear.

Regarding the endoscopic and nonendoscopic therapies, EVL was the most common therapeutic endoscopic approach for EVH (84.3%) and had an in-hospital mortality of 12.3%, the lowest mortality of all variceal localizations and all endoscopic and nonendoscopic treatments in our study. This is in line with the current guidelines, recommending EVL as the first-line treatment for EVH, and therefore reflects the current clinical practice in Germany and may be further explained by the highest level of experience in using this technique [7, 21]. The injection of vasoactive substances and fibrin glue (8.1%) and EST (2.7%) were the less frequently applied techniques in EVH, both with significantly higher mortality (30.8% and 20.5%, respectively). The German coding system does not differ between injecting vasoactive substances (e.g., suprarenin) and fibrin glue. Because the injection of vasoactive substances in AVH is not the SOC, we assume that most of the cases of injection represent the actual cases of fibrin glue injection/EST. Still, in the end, this point remains arguable. Endoscopic tamponade (Sengstaken-Blakemore tube) and esophageal stent application were rarely used techniques (combined: 3.5% of all endoscopic therapies) with significantly higher in-hospital mortality (tamponade: 65.9%; stent: 63.5%) compared to EVL. An esophageal tamponade is an option for uncontrolled AVH associated with hemodynamic instability or failure of endoscopic treatment as temporizing treatment with slightly lower reported mortality (42%) compared to our data, but the data are from single-center studies and heterogeneous [57, 62].

Combined endoscopic therapies were used significantly less often than in GVH, which may reflect more effective primary hemostasis in EVH by EVL with less need for subsequent therapies. To our knowledge, there are no systematic data about combination/different therapy modalities regarding the bleeding site. Overall, there was significantly higher in-hospital mortality in combination therapies (30.5%) compared to single-modality therapies (16.1%). Endoscopic combination therapy in AVH is not the SOC and usually represents treatment failure of the primarily used modality. The failure rate of EVL was reported previously to be around 10% [63, 64], but we could not identify the reasons for individual treatment failures due to the data structure. The predominantly used combined approach in EVH in our study was a combination of EVL + injection followed by EVL + EST. Other studies reported EST as the most frequent subsequent therapy after EVL followed by Re-EVL [64]. Overall, EVL was the most common combination therapy in our study and was used as a combination partner in 84.3% of the cases. Interestingly, there were no differences regarding the in-hospital mortality between EVL + injection (32.5%) vs. EVL + clipping (31.9%) vs. EVL + EST (29.9%). This finding possibly emphasizes the effectiveness of EVL in EVH. These data need further investigation since extensive studies about subsequent therapies in EVH are missing. We have to state that we could not differ between the first and subsequent therapy, but we assume that injection/EST was reserved for primary EVL failure. Thus, this remains speculative. EVL + TIPS (21.2%) and EVL + embolization (15.3%) had a lower in-hospital mortality compared to every other (endoscopic) therapy in combination with EVL. On the other hand, non-EVLs, including combination therapies (e.g., EST + embolization), were associated with increased mortality in EVH. These findings underline the potential role of early TIPS after failed primarily endoscopic therapy as a rescue option in AVH and potentially preemptive or in secondary prevention as well [65, 66].

The overall mortality in GVH in our study was 19.1% and significantly higher than in EVH (16.1%). Gastric varices can be classified using analog Sarin classification into gastroesophageal varices type 1 (GOV-1; esophageal varices extending below the cardia, 75% of gastric varices), cardiofundal varices (GOV-2), and isolated gastric varices type 1 and 2 (IGV-1 and IGV-2) [67]. GOV-1 is the most common bleeding site, but AVH from GOV-2 is often more severe and more difficult to control and shows a higher risk of recurrent bleeding and mortality (up to 45%) [7]. Unfortunately, the German coding system does not allow to discriminate between the different types of gastric varices. Therefore, we could not perform subgroup analyses using analog Sarin classification. In GVH, injection and EST accounted together for 71.5% of all therapies in our study, representing the guideline recommendations and the current SOC as injection of cyanoacrylate/glue (EST) and EVL are accepted options for endoscopic therapy in patients bleeding from gastric (cardiofundal) varices as both therapies are equally effective in primary hemostasis. Still, injection/EST showed lower rebleeding rates [25, 68], and EVL should only be performed on small gastric varices where the complete vessel can be suctioned into the ligation device [7]. In our study, EVL was performed in 583 cases of gastric variceal bleeding. There was a trend toward lower mortality in EST vs. EVL (EST: 14%; EVL: 16.3%), but the results failed to show significance. The mortality rates for EST and EVL in GVH in our data are in line with the previously published data, reporting mortality rates between 10% and 30% [69, 70].

TIPS was performed in 1502 cases, and radiologic intervention (embolization) was performed in 511 cases. TIPS in AVH overall had an in-hospital mortality of 19.2%, which is slightly higher than our study's overall mortality. This finding is in line with the recently published data regarding TIPS [71].

Finally, there was a significant difference concerning the annual number of cases per hospital and in-hospital mortality, with the highest in-hospital mortality in low-volume centers. There was no difference between the other groups (high-volume vs. medium-high vs. medium-low centers). We assume that the short-term (in-hospital) outcome of AVH does not depend on the number of cases per hospital per se as long as specific requirements (generally available medication such as PPI, antibiotics, an endoscopic department, certain level of endoscopic skills (EVL, EST) to achieve primary hemostasis, and intensive care unit) are maintained with high probability of immediate termination of bleeding at least for the vast majority of cases and subsequent endoscopic therapies and multiprofessional approaches (TIPS and radiologic interventions) can follow secondary in selected cases. To our knowledge, there are no data regarding the hospital size and in-hospital mortality in AVH yet.

We acknowledge some important limitations of our study. First, a retrospective database evaluation cannot draw any causal link between the observed comorbidities, the performed endoscopic therapies, or the reported proportions, especially against the background that we were not able to discriminate between bleeding and nonbleeding lesions and other individual circumstances (e.g., medication, laboratory values, local SOC, the stadium of liver function deterioration, portal vein thrombosis, cardiovascular diseases, and other malignant diseases), which may contribute to the observed results. This is particularly the case for the Child–Pugh classification. Grading analog Child–Pugh has been shown to play a significant role in assessing the risk of bleeding (and rebleeding) in cirrhotic patients [72]. The German Diagnosis-Related Groups (DRG) system has taken the grading analog Child–Pugh classification just recently into account; therefore, it was not recorded systematically in the past, and we could not stratify our results by the Child–Pugh classification.

Furthermore, our data do not reveal essential parameters such as pretreatment/subsequent therapies, readmissions, or mid- and long-term follow-up after hospital discharge. It is also important to note that we could not determine the intention to treat (e.g., primarily vs. secondary prevention and the rebleeding rate), and we must state that we could not record the number of performed therapies in one patient. Thus, the proportion of therapies is possibly overestimated, and the increased mortality in the treatment group does not reflect a causal relation and is highly probable due to more severe cases of AVH (e.g., active and rebleeding), which may lead to endoscopic therapies in these cases. Furthermore, we could not discriminate between different sides of GVH and other sides of AVH (e.g., rectal). Finally, no information on coding quality in Germany is available, and the database is not subject to systematic quality control between individual hospitals [73–75]. Nevertheless, it can be considered that endpoints such as death are little or not influenced by coding errors and may correctly represent medical practice.

## 5. Conclusion

Our study gives a detailed insight into the incidence, patient-related risk factors, endoscopic therapies, and overall in-hospital mortality, as well as regional differences in a large AVH collective in Germany. Furthermore, we were able to define mortality-associated complications and their impact. Our present findings should trigger prospective randomized studies with proper methodological designs for studying the management of AVH.

## Figures and Tables

**Table 1:** Characteristics of the study population, adverse events.

Variable	Study population
Total number of AVH cases	65,357
Female (number and percentage)	20,725 (31.7%)
Male (number and percentage)	44,632 (68.3%)
Age (mean, SD)	60.3 (± 12.9)
Age group (number and percentage)	
0–17 years	172 (0.3%)
18–30 years	533 (0.8%)
31–50 years	13,923 (21.3%)
51–70 years	35,371 (54.1%)
> 70 years	15,358 (23.5%)
Comorbidities (number and percentage)	
Alcoholic fibrosis and cirrhosis	39,589 (60.6%)
Other fibrosis and cirrhosis	20,385 (31.2%)
Hepatic cancer	1643 (2.5%)
Other liver diseases	3740 (6.6%)
Adverse events	
Bleeding anemia	39,285 (60.1%)
Hypovolemic shock	8373 (12.8%)
Sepsis	2466 (3.8%)
Acute kidney injury	5486 (8.4%)
Hepatorenal syndrome	5866 (9%)
Hepatic encephalopathy	11,956 (18.3%)
Ascites	27,066 (41.4%)
Artificial ventilation	10,636 (16.3%)
Duration of artificial ventilation	
1–48 h	5346 (50.3%)
49–168 h	2766 (26%)
> 168 h	2524 (23.7%)
ARDS	444 (0.7%)

Abbreviations: ARDS = acute respiratory distress syndrome, AVH = acute variceal hemorrhage, SD = standard deviation.

**Table 2:** Characteristics of in-hospital mortality, cases per year, and geographical distribution of cases.

Variable	Study population
Total number of AVH cases	65,357
Overall in-hospital mortality, 2010–2019 (percentage)	18.6%
In-hospital mortality by year	In-hospital mortality, percentage(total number of cases)
2010	19.3% (6440)
2011	18.7% (6656)
2012	18.2% (6479)
2013	19% (6605)
2014	17.8% (6517)
2015	18.6% (6645)
2016	18.4% (6749)
2017	18.9% (6555)
2018	18.7% (6320)
2019	18.1% (6391)
Annual number of AVH cases per hospital (2010–2019)	In-hospital mortality, percentage (total number of cases)
1–5 (LVC)	20% (12.797)
6–9 (MLVC)	17.8% (14.260)
10–13 (MHVC)	18.1% (11.630)
≥ 14 (HVC)	18.5% (26.670)
MLVC vs. LVC	OR 0.78 (95% CI: 0.72–0.85), *p* < 0.001
MHVC vs. LVC	OR 0.77 (95% CI: 0.71–0.84), *p* < 0.001
HVC vs. LVC	OR 0.58 (95% CI: 0.54–0.62), *p* < 0.001
Incidence rate (per 100,000 residents/year)	
Nationwide	8.9
New Federal States (without Berlin)	10.4
Old Federal States (including Berlin)	8.2
City states (Berlin, Bremen, Hamburg)	10.5
Federal state	Total number, percentage (incidence rate per 100,000 residents/year)
Baden-Württemberg	8327 (12.7%; 7.7)
Bavaria	9411 (14.4%; 7.4)
Berlin	3715 (5.7%; 10.6)
Brandenburg	2402 (3.7%; 9.7)
Bremen	702 (1.1%; 10.5)
Hamburg	1848 (2.8%; 10.3)
Hesse	4094 (6.3%; 6.7)
Mecklenburg–West Pomerania	1691 (2.6%; 10.5)
Lower Saxony	5473 (8.4%; 7)
North Rhine–Westphalia	12,897 (19.7%; 7.3)
Rhineland-Palatinate	2933 (4.5%; 7.3)
Saarland	928 (1.4%; 9.3)
Saxony	4496 (6.9%; 11)
Saxony-Anhalt	2288 (3.5%; 10.2)
Schleswig-Holstein	1806 (2.8%; 6.3)
Thuringia	2346 (3.6%; 10.8)

Abbreviations: AVH = acute variceal hemorrhage, CI = confidence interval, HVC = high-volume centers, LVC = low-volume centers, MHVC = medium–high volume centers, MLVC = medium–low volume centers, OR = odds ratio.

**Table 3:** Characteristics of the study population and therapies.

Variable	Study population
EVH + GVH, treatment, total number	56,897
Endoscopic therapies, total number	54,884
TIPS	1502
Embolization (radiol.)	511

Treatment (modality)	Number (mortality percentage)

EVH, endoscopic treatment, total number (mortality percentage)	46,835 (16.1%)
Ligation	39,499 (12.3%)
Injection (vasoactive and fibrin glue)	3790 (30.8%)
EST	1276 (20.5%)
Tamponade	1283 (65.9%)
Clipping	614 (27.9%)
Stent	373 (63.5%)
EVH, combination therapies, total number	6481 (30.5%)
EVL + injection	2126 (32.5%)
EVL + clipping	586 (31.9%)
EVL + EST	1868 (29.9%)
EVL + TIPS	722 (21.2%)
EVL + embolization	163 (15.3%)
Injection + clipping	469 (39.7%)
Injection + EST	294 (32.3%)
EST + clipping	140 (37.9%)
EST + TIPS	99 (26.3%)
EST + embolization	14 (35.7%)
GVH, treatment, total number (mortality percentage)	8049 (19.1%)
Injection (vasoactive and fibrin glue)	3199 (22.1%)
EST	2564 (14%)
Clipping	1571 (23.4%)
EVL	583 (16.3%)
Absorbent substances	132 (37.9%)
GVH, combination therapies, total number (mortality percentage)	1342 (21.8%)
Injection + clipping	524 (24.6%)
Injection + EST	468 (16.9%)
Injection + EVL	53 (22.6%)
Injection + absorbent substances	24 (37.5%)
EST + clipping	134 (17.2%)
EST + embolization	25 (28%)
EST + TIPS	114 (28.9%)

Abbreviations: EST = endoscopic sclerotherapy, EVL = endoscopic variceal ligation, EVH = esophageal variceal hemorrhage, GVH = gastric variceal hemorrhage, TIPS = transjugular intrahepatic portosystemic shunt.

**Table 4:** Sex and organ complications are associated with increased in-hospital mortality (univariate and multivariate Cox regression analyses).

Parameter	Univariate		Multivariate	
	OR (95% CI)	*p* value	OR (95% CI)	*p* value
Age (per year)	1.003 (1.001–1.004)	0.001	1.02 (1.02–1.03)	< 0.001
Female vs. male	0.89 (0.86–0.93)	< 0.001	0.91 (0.86–0.96)	0.001
Hypovolemic shock	5.98 (5.7–6.28)	< 0.001	2.76 (2.57–2.96)	< 0.001
HRS	7.28 (6.88–7.7)	< 0.001	4.15 (3.83–4.49)	< 0.001
AKI	6.87 (6.48–7.27)	< 0.001	2.59 (2.4–2.79)	< 0.001
Sepsis	9.11 (8.37–9.92)	< 0.001	2.19 (1.94–2.46)	< 0.001
Ascites	2.14 (2.05–2.23)	< 0.001	1.16 (1.09–1.22)	< 0.001
HE	3.32 (3.17–3.47)	< 0.001	4.15 (3.83–4.49)	< 0.001
Artificial ventilation	10.87 (10.38–11.39)	< 0.001	6.15 (5.76–6.57)	< 0.001
ARDS	17.74 (14.06–22.38)	< 0.001	2.58 (1.94–3.44)	< 0.001

Abbreviations: AKI, acute kidney injury; ARDS, adult respiratory distress syndrome; HE, hepatic encephalopathy; HRS, hepatorenal syndrome.

## Data Availability

The results of the remote data analyses provided by the Federal Statistical Office are available from the corresponding author upon reasonable request. Contact: Dr. med. Alexander Mertens; E-Mail: alexander.mertens@med.uni-duesseldorf.de.
